# Improving power in PSA response analyses of metastatic castration-resistant prostate cancer trials

**DOI:** 10.1186/s12885-022-09227-7

**Published:** 2022-01-26

**Authors:** Michael J. Grayling, Martina McMenamin, Robert Chandler, Rakesh Heer, James M. S. Wason

**Affiliations:** 1 Population Health Sciences Institute, Newcastle University, Baddiley-Clark Building, Richardson Road, Newcastle upon Tyne, NE2 4AX UK; 2 WHO Collaborating Centre for Infectious Disease Epidemiology and Control, School of Public Health, Li Ka Shing Faculty of Medicine, The University of Hong Kong, Hong Kong, Special Administrative Region China; 3 Northern Centre for Cancer Care, Newcastle upon Tyne, UK; 4 Translational and Clinical Research Institute, Newcastle University Centre for Cancer, Newcastle University, Newcastle upon Tyne, UK; 5 Department of Urology, Freeman Hospital, The Newcastle upon Tyne Hospitals NHS Foundation Trust, Newcastle upon Tyne, UK

**Keywords:** Augmented binary, Biochemical response, Composite endpoint, Phase II cancer trial, Responder analysis, Statistical analysis

## Abstract

**Background:**

To determine how much an augmented analysis approach could improve the efficiency of prostate-specific antigen (PSA) response analyses in clinical practice. PSA response rates are commonly used outcome measures in metastatic castration-resistant prostate cancer (mCRPC) trial reports. PSA response is evaluated by comparing continuous PSA data (e.g., change from baseline) to a threshold (e.g., 50% reduction). Consequently, information in the continuous data is discarded. Recent papers have proposed an augmented approach that retains the conventional response rate, but employs the continuous data to improve precision of estimation.

**Methods:**

A literature review identified published prostate cancer trials that included a waterfall plot of continuous PSA data. This continuous data was extracted to enable the conventional and augmented approaches to be compared.

**Results:**

Sixty-four articles, reporting results for 78 mCRPC treatment arms, were re-analysed. The median efficiency gain from using the augmented analysis, in terms of the implied increase to the sample size of the original study, was 103.2% (IQR [89.8,190.9%]).

**Conclusions:**

Augmented PSA response analysis requires no additional data to be collected and can be performed easily using available software. It improves precision of estimation to a degree that is equivalent to a substantial sample size increase. The implication of this work is that prostate cancer trials using PSA response as a primary endpoint could be delivered with fewer participants and, therefore, more rapidly with reduced cost.

**Supplementary Information:**

The online version contains supplementary material available at 10.1186/s12885-022-09227-7.

## Background

While recent advances have considerably reduced the number of men who die from prostate cancer (PC), it remains the second-most common form of death from cancer in the USA and UK [1]. There thus remains an urgent need for better treatments, in particular for men who present with advanced PC. In trials of treatments for advanced disease, the main outcome of interest is typically overall survival (OS). In many instances though, particularly for phase II metastatic castration-resistant PC (mCRPC) trials, an alternative outcome that can be observed more quickly is required. Serving this purpose, prostate-specific antigen (PSA) is a serum biomarker that can be measured easily, with changes in its level having been shown to correlate with OS [2–4]. Changes in PSA are typically evaluated by comparing continuous PSA change to a specified threshold; forming a binary ‘PSA response’ variable. PSA response is routinely used as a primary or secondary endpoint in advanced disease PC trials, and it has been shown to be a potential surrogate for OS in a study of 22 trials [4].

Several recommendations on what level of change in PSA is clinically meaningful have appeared in the literature; Scher et al. [5] provide an overview of these. A ≥ 50% reduction in PSA from baseline was recommended based on retrospective studies showing this was associated with increases in survival. A ≥ 30% reduction was proposed using evidence from randomised trials [6]. The first Prostate Cancer Clinical Trials Working Group (PCWG1) recommended defining PSA response as a ≥ 50% decrease from baseline [7]. This was updated in the PCWG2 guidance [8] to suggest avoiding reporting PSA response rates and instead provide waterfall plots of PSA change. PCWG2 recommended PSA progression as an endpoint, defined as a 25% increase in PSA. These recommendations were retained in the PCWG3 guidance [9].

Regardless of threshold choice, PSA response (with, e.g., a ≥ 30% decline threshold) like other ‘responder’ endpoints is analysed as a binary outcome. Analyses focus on the proportion of patients classified as responders, without consideration of the actual PSA change. A patient with a 31% reduction is treated the same as someone with a 90% reduction, but completely differently from someone with a 29% reduction. In practice the patients with 31 and 29% reductions are likely more similar than the 31 and 90% patients.

This illustrates the issue with dichotomisation of continuous measures: it discards information and thereby leads to reductions in power [10–12]. To address this, there are ‘augmented’ methods available that can increase efficiency [13–15]. The main advantage of these methods is that one can typically estimate the proportion of responders more precisely; the underlying continuous data being exploited to improve evaluation on the simpler responder outcome. Previous work has shown the efficiency gained is often equivalent to increasing the sample size by at least 30%, without needing extra data to be collected.

Due to the availability of waterfall plots in PC trial reports, it is possible to extract continuous PSA change data. We set the objective of systematically doing this to show how PSA response analyses compare between augmented and traditional methods. We demonstrate how the augmented analysis would considerably increase the efficiency of PSA response analyses. We provide a case study to clarify the value of this approach further and conclude by commenting on what our findings may mean for PC trials.

## Methods

### Identification and extraction of prostate-specific antigen change datasets

For simplicity, we restricted attention to:

- PSA response endpoints consisting of whether a single continuous outcome (change from baseline; either best change or at a specified time post-randomisation) is above a threshold (e.g., 50% reduction).
- Where a PSA response rate needs to be estimated on an arm-by-arm basis.

However, as discussed further later, the augmented method is applicable more generally; this includes to both comparison of response rates by arm in a randomised trial, and to more complex forms of responder endpoint.

We wished to identify published PC trial reports that included waterfall plots of PSA data, such that the original dataset could be reverse-engineered for re-analysis. Given the important role PSA response rates have in mCRPC settings, we planned to focus our analyses on trials in this domain. However, to provide as broad an evaluation as possible, we also sought waterfall plots in PC trial reports of other disease stages.

We searched PubMed Central using “PSA AND waterfall” on October 12 2019. This returned 280 articles, which were pre-screened by MJG to identify those that contained a waterfall plot in which the y-axis indicated PSA change data was presented; 154 articles passed this pre-screening. Ten remaining articles were then randomly selected for replicate pilot evaluation for inclusion and data extraction by JMSW, MJG, and MMM. The inclusion criteria was: presents a waterfall plot of clinical trial data for which automated data reverse-engineering could be applied (see below). For each of the ten pilot articles deemed eligible for inclusion, data for the following items were extracted by each reviewer:

1. Dichotomisation threshold (e.g., 30% decrease).
2. Number of patients assumed in the analysis.
3. Number of responses assumed in the analysis.
4. Reported point estimate for the PSA response rate.
5. Reported confidence interval (CI) for the PSA response rate.
6. Reverse-engineered PSA change data, as extracted using WebPlotDigitizer [16]. Note this tool in general provides high precision in data reverse-engineering, but some small inaccuracies are unavoidable. We discuss later sensitivity analyses performed to assess the impact of any inaccuracies.
7. Disease population (e.g., mCRPC).
8. Phase of research (e.g., phase II).

The three reviewers agreed for all ten pilot articles on whether they met the inclusion criteria. The piloting revealed a number of waterfall plots clipped the presentation of data at an upper percentage increase. To enable a sensitivity analysis to be performed to what the true values may have been, two additional items for data extraction were then added:

9. Number of clipped bars.
10. Clip point.

Some small differences in extracted data for the included articles in the pilot evaluation were present. However, the reasons for these differences were easily determined and therefore the remaining 144 articles were randomly allocated for single review between JMSW, MJG, and MMM. More details on the nine extraction items and on the differences observed in the pilot review are given in the Supplementary Materials.

Following completion of data extraction, MJG reviewed each of the articles for which the reverse-engineered dataset (Item 6) did not match the data extracted for Items 1–5 and 9–10, to establish why this was the case. A small number of differences were present due to typographical errors. The majority of differences were due to trials in which an intention-to-treat analysis was performed but waterfall data was only available for a subset of patients. Note that given such differences, along with the presence of bar clipping in several waterfall plots and the minor but inevitable inaccuracies in the reverse-engineered continuous data, our analyses should not be interpreted as definitive re-analyses of the included trials. They instead represent a realistic evaluation of the efficiency gains that may be attained when using the augmented analysis approach for data with distributions highly similar to those observed in practice.

### Dataset re-analysis

#### Notation

The final outcome of data extraction was a set of PSA change from baseline datasets along with their dichotomisation thresholds. We now describe how we re-analysed these datasets to compare standard and augmented analyses.

In a given reverse-engineered dataset, denote the percentage reduction in PSA level for patient *i* by *Y*_*i*_. We assume patient *i* is classified as a responder if *Y*_*i*_ > *d*, where *d* is the dichotomisation threshold matching the chosen definition of PSA response. The responder outcome is *S*_*i*_: it takes the value 1 if patient *i* is a responder and 0 if they are a non-responder. Thus, *S*_*i*_ = 1 if *Y*_*i*_ > *d* and *S*_*i*_ = 0 otherwise. Our objective was then to compare methods of inference for the PSA response rate *p* = Prob(*S*_*i*_ = 1).

#### Standard analysis method

Standard methods analyse the *S*_*i*_, treating them as binary. The estimate of *p* is $\hat{p}={\sum}_{i=1}^n{S}_i/n$, with *n* the sample size. To compute a CI for *p*, there are many available approaches. We use Clopper-Pearson, as this is a standard option for which software is readily accessible.

#### Augmented method

The augmented method assumes the *Y*_*i*_ are normally distributed. The first step therefore ensures this assumption is met as closely as possible through data transformation. We use a Box-Cox transform, which creates a variable of the form ${Z}_i={Y}_i^{\lambda }/\lambda$, with *λ* chosen so that *Z*_*i*_ is as close to normality as possible. We also transform the dichotomisation threshold using the same *λ*, *d*_*λ*_ = *d*^*λ*^/*λ*, so that the definition of responder remains *S*_*i*_ = 1 if *Z*_*i*_ > *d*_*λ*_ and *S*_*i*_ = 0 otherwise.

We find the best-fitting normal distribution to the values *Z*_1_, …, *Z*_*n*_. If the normal distribution is represented by *N*(*μ*, *σ*^2^), this allows the delta-method to be used to get the variance of $1-\Phi \left(\frac{d_{\lambda }-\mu }{\sigma}\right)$, which is the estimated probability of a response, $\hat{p}$. We form a CI for $\hat{p}$ in this case using Wald’s approach.

#### Method comparison

Our re-analysis of the reverse-engineered datasets provided point estimates and 95% CIs by arm when using the standard and augmented analyses. To evaluate the efficiency gain provided in each case from using an augmented analysis, we:

1. Compare the width of the 95% CIs: The percentage change in the 95% CI width is 100(*l*_st_ − *l*_aug_)/*l*_st_, where *l*_st_ and *l*_aug_ are the widths of the 95% CIs returned by the standard and augmented analyses.
2. Compute the implied increase in the sample size from using the augmented analysis: For the point estimate $\hat{p}$ estimated using the standard method, we determine how large the sample size of the trial would have had to have been using the standard analysis to achieve the 95% CI width provided by the augmented analysis. If the trial’s actual sample size is *n*, and the implied sample size for a 95% CI width of *l*_aug_ is *n*_imp_, we present the percentage increase 100(*n*_imp_ − *n*)/*n*.

#### Sensitivity analyses

To determine the impact of clipped bars or inaccuracies in the reverse-engineered data, sensitivity analyses were performed varying the extracted continuous data. These are described in the Supplementary Materials; they indicate the augmented approach is robust to the underlying continuous data.

#### Software

Data and code to replicate our analyses is available from https://github.com/mjg211/article_code. An R Shiny application that compares the two analysis approaches for a given dataset is provided at https://martinamcm.shinyapps.io/psaresp/. A demonstration of this application is given in the Results.

## Results

### Included articles

**Fig. 1:**
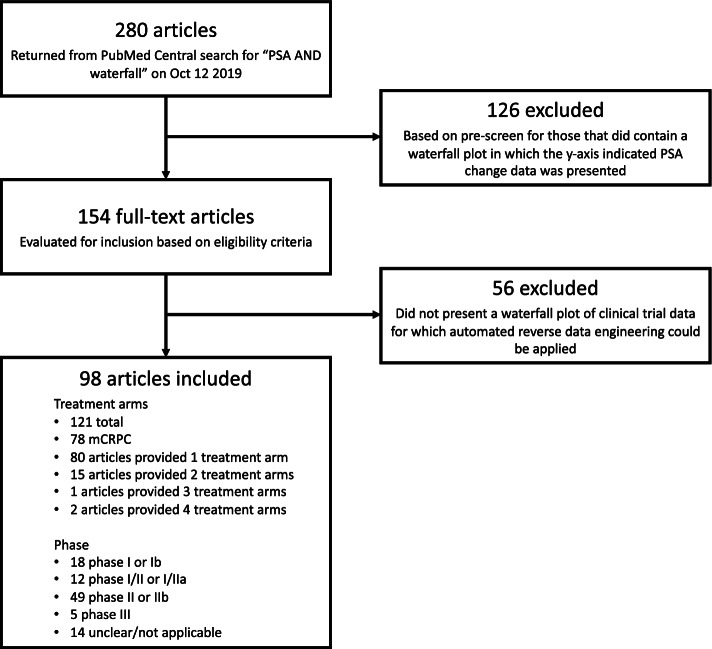
Identification of relevant datasets following initial PubMed Central search

Ninety-eight articles reporting results for 121 treatment arms were identified for which re-analysis could be performed, including 64 articles reporting 78 mCRPC treatment arms (Fig. 1). Fifty-percent (49/98) of the articles presented results of phase II research; we comment on this in the Discussion in relation to the applicability of the augmented-binary method.

Here, we present results for the re-analysis of the 78 mCRPC reverse-engineered datasets, which together account for a re-analysis of data from 2664 patients (median *n* per dataset = 18, IQR [26.5,45.75]). The Supplementary Materials provides additional analyses that demonstrate results are similar across the mCRPC and non-mCRPC data.

### Comparison of standard and augmented analysis approaches

**Fig. 2:**
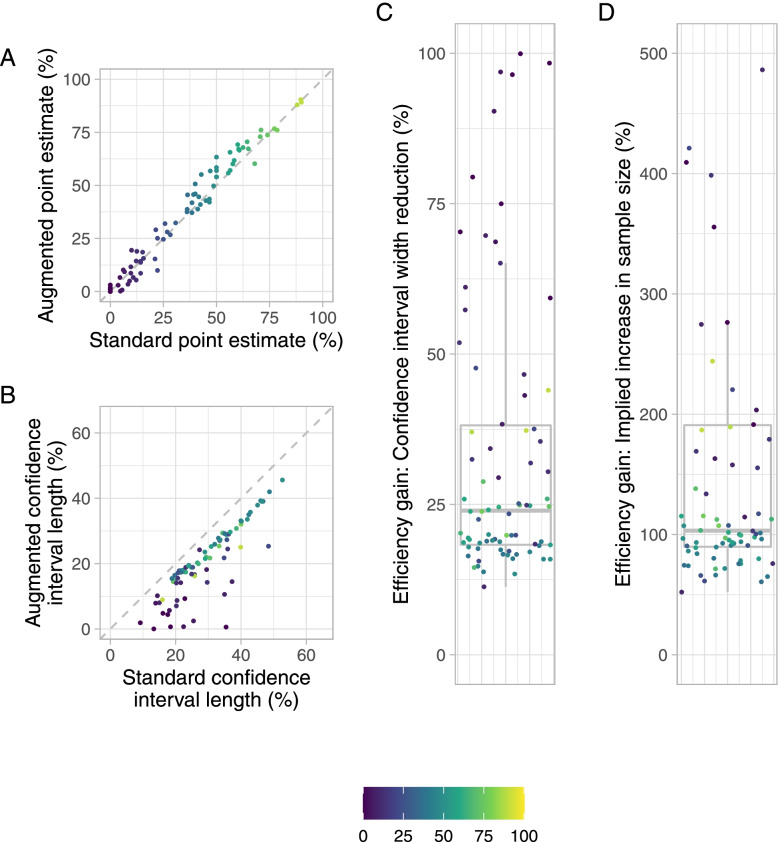
Comparison of the standard and augmented analysis approaches for the 78 included mCRPC datasets. Points are shaded according to the value of the standard point estimate. **A**: The standard and augmented point estimated are compared. **B**: The width of the standard and augmented point estimates are compared. **C**: The efficiency gains, in terms of the percentage confidence interval width reduction, are given. **D**: The efficiency gains, in terms of the percentage increase to the trial’s sample size, are given. For **D**, the limits are constrained to [0,500] for aesthetic purposes; 9 trials for which substantially larger efficiency gains were observed are omitted from this sub-figure

Standard and augmented point estimates and 95% CIs were computed and compared for each of the 78 datasets (Fig. 2). As expected, and as would be desired on-average, the difference between the point estimates was often small (Fig. 2A); the median difference (augmented minus standard point estimate) was 1.6% (IQR [− 0.8,4.9%]) and the Pearson correlation between the two estimates was 0.98.

In all 78 datasets, the augmented analysis returned a 95% CI with a narrower width (Figs. 2B-C). The median efficiency gain from using the augmented analysis, in terms of the percentage reduction in the width of the 95% CI for the response rate, was 24.0% (IQR [18.3,38.1%]). In terms of the implied percentage increase to the original sample size (Fig. 2D), the median efficiency gain was 103.2% (IQR [89.8,190.9%]). That is, the augmented analysis approach improved precision on average to a degree equivalent to a 103.2% increase to the trial sample size.

Note that the cases with extreme increases in efficiency are typically those in which the standard point estimate was small. This is a result of the fact that the standard 95% CI is often then far wider than it need be when the PSA continuous change data is far from the response threshold.

### Case study: Hofman et al.

Hofman et al. [17] report on a single-centre single-arm phase II trial of patients with mCRPC and progressive disease after standard therapy. Eligible patients received up to four cycles of intravenous [^177^Lu]-PSMA-617, a radiolabelled small molecule, at six weekly intervals. Their primary endpoint was PSA response, defined as a ≥ 50% PSA decline from baseline. This was ultimately confirmed in 17/30 patients; thus the performed standard analysis led to a reported point estimate for PSA response of 56.7% [95% CI (37.4,74.5%)].

**Fig. 3:**
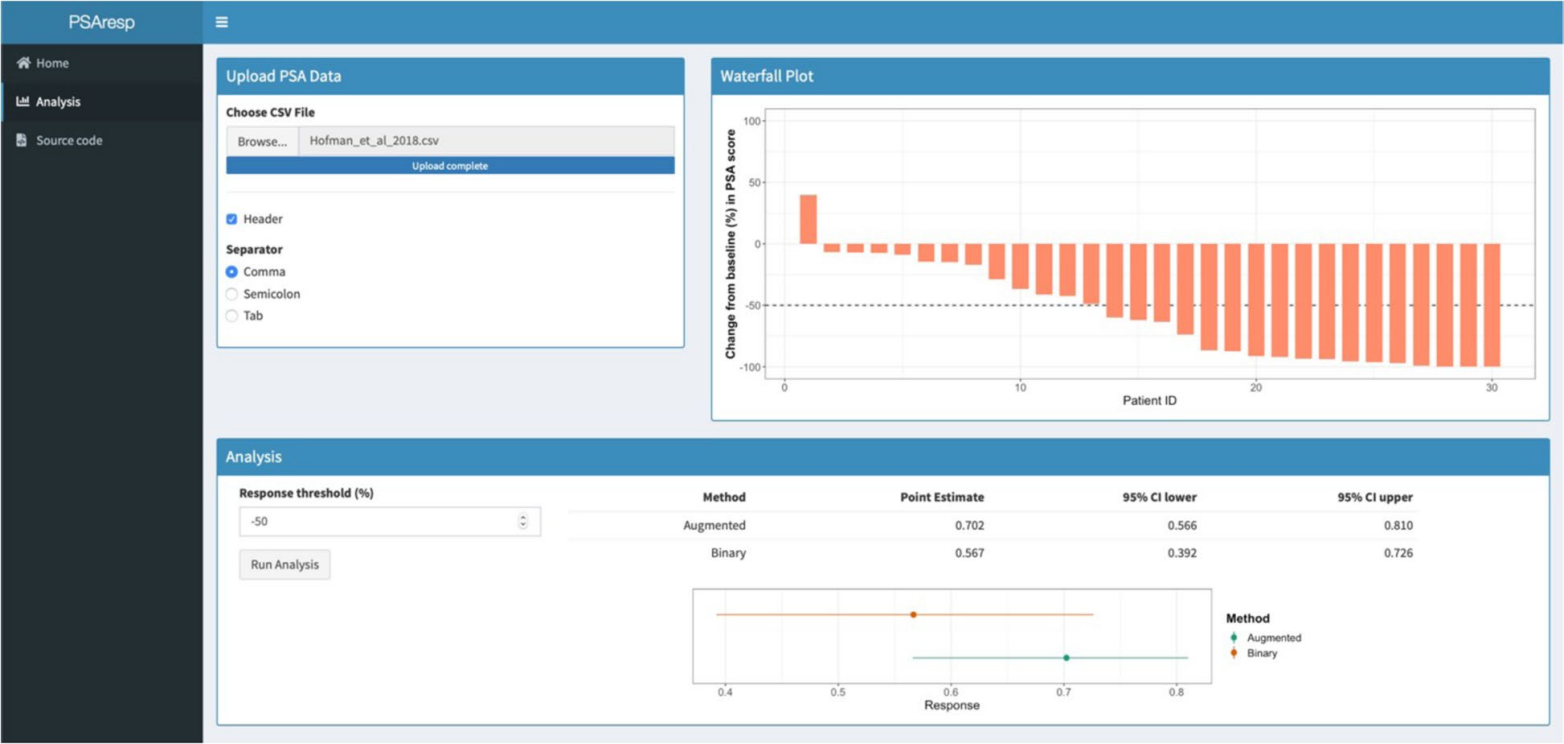
Comparison of the standard and augmented analysis approaches, for the case study Hofman et al. [17], using the online web application. A re-created waterfall plot can be seen, along with the computed point estimate and confidence interval for the two analysis approaches after response threshold selection

We re-analyse with the augmented approach to expand on how it compares with the standard analysis. Figure 3 shows a screenshot of the online application for comparing the two analyses. Data is uploaded, in this case that reverse-engineered from the waterfall plot in Hofman et al. [17], and the application produces its own waterfall plot. It is easy to see why the continuous data can improve the analysis; there is a wide spread in the continuous values and as discussed earlier it is illogical to treat the patient with an approximately 49% decline in PSA the same as that who experienced an increase in PSA.

Here, the augmented point estimate is 70.2%, substantially larger than the standard given above; this is a consequence of the distribution of the underlying continuous data, where many patients experienced close to a 100% decline. Use of the continuous data in the augmented analysis results in a 95% CI of (56.6,81.0%); a reduction in width of 26.9% over the standard CI. This translates to an implied increase to the sample size of the trial of 129.0%.

## Discussion

Conventional methods of analysis for PSA response are statistically inefficient. Our re-analysis of 78 mCRPC trial datasets established a median 24.0% reduction in the width of the 95% CI for the PSA response rate could have been possible through an augmented analysis approach. This translated to a median efficiency gain in terms of the implied percentage increase to the sample size of the trials of 103.2%. This augmented methodology requires no additional data to be collected and can be implemented easily; we demonstrated this implementation for a particular case study using an online application.

With its potential advantages clear, important questions are then evident in relation to when the augmented analysis is statistically valid and when it may be most applicable in practice. To date, the augmented analysis approach has been demonstrated to be statistically robust in several simulation studies for oncology settings [18, 19]. It has also been applied in reanalyses of real datasets in rheumatoid arthritis [20] and lupus [21] and shown to provide substantially increased power without inflation in the type I error-rate. It is applicable to evaluation of treatment effects on a single arm or for the comparison of effects between arms, while it can also be applied to more complex responder endpoints than that considered here (where we focused on PSA response endpoints consisting of whether a single continuous outcome was above a threshold). The main assumption made is that the underlying continuous outcome data is normally distributed. The results can be sensitive to this assumption [22], although it is possible to transform outcome data to better be approximated by a normal distribution. The augmented analysis method has always demonstrated improved power for responder outcomes measured at a fixed timepoint, although this is not so consistent for time-to-event outcomes [23]. Its main disadvantage is its increased computational requirements, especially when there are multiple timepoints [19], or it is a complex responder outcome [21]. Because of the additional assumptions made, the method may be more suitable for earlier phase research, or secondary analyses of phase III trials; it may not be accepted as the primary analysis in a confirmatory trial setting.

We acknowledge again limitations to our work. Due to the process of data reverse-engineering, the presence of bar clipping in 42/121 extracted datasets, and the fact published waterfall plots may only include data for a subset of enrolled patients, our re-analyses should not be considered a definitive re-assessment of the results from included trials. However, our work does reflect a comprehensive evaluation of the level of efficiency gain that may be possible with the augmented analysis approach on data highly similar to that accrued in practice.

We end with a discussion of what our work may mean for the reporting of PSA response rates in PC trial reports. A principal motivator for our work was to assess the utility in practice of the augmented analysis. In this sense, examining PSA response data specifically is based on convenience, given the frequency with which it is available in published reports. It is not meant as a recommendation that such analyses should be performed in contradiction to PCWG3 guidance. Nonetheless, we argue that in recommending such data be presented in waterfall plots, there is a tacit indication in the PCWG3 guidance of the value of the continuous data. Furthermore, 96/98 (98.0%) articles in our re-analysis reported a PSA response rate (as opposed to simply presenting waterfall data). Thus, it appears PSA response rates are still routinely reported alongside waterfall data in PC trial reports, indicative of the PC community finding value in them. Whenever such response rates are reported, there is an ethical imperative to utilise patient data as effectively as possible. Consequently, we strongly recommend utilising the augmented approach. Finally, we highlight that the augmented methodology described here is one implementation of a more flexible framework. It could be readily applied to the analysis of, e.g., time to PSA progression, which was recommended in PCWG3.

## Conclusions

In conclusion, the augmented analysis can provide substantial statistical advantages. Given its ease of use, it offers an effective means of improving the efficiency of clinical trials that utilise responder endpoints, such as PC trials that analyse PSA response or time to PSA progression. Embracing the use of this method could help make clinical trials far more efficient, reducing the sample size required by clinical trials, which will in turn speed up research and reduce costs. For fields in which the clinical landscape evolves rapidly, this may be invaluable to maximizing the value of a given clinical trial.

## Supplementary Information

**Additional file 1.**

## Data Availability

The data and R code supporting the conclusions of this article are freely available at https://github.com/mjg211/article_code.

## Abbreviations

CI: Confidence Interval
mCRPC: Metastatic Castration-Resistant Prostate Cancer
PC: Prostate Cancer
PCWG: Prostate Cancer Working Group
PSA: Prostate Specific Antigen
