# Supplementary material for: Improving power in PSA response analyses of metastatic castration-resistant prostate cancer trials
Source: BMC Cancer. 2022 Jan 26;22:111. doi: 10.1186/s12885-022-09227-7 (PMC8793251; doi:10.1186/s12885-022-09227-7)
Supplement: Supplementary file 1 — Additional file 1. [file 12885_2022_9227_MOESM1_ESM.docx]

**Additional file 1**

Additional information on “Identification and extraction of prostate-specific antigen change datasets”

The following ten data items were extracted for all articles deemed eligible for inclusion:

1. Primary dichotomisation threshold (PDT): Many studies reported findings for multiple thresholds (e.g., 30% and 50% decrease). Only the PDT to be used in our re-analysis was extracted. In two studies, where no PDT was given and only waterfall data was presented, a 50% decrease in PSA was used in the comparison of the standard and augmented methods as this was the most common PDT across all other included articles.
2. Number of patients assumed in the analysis for the PDT: The number of patients reported in the article that were assumed for the analysis of the data for the PDT, by arm. This was extracted for comparison with the reverse engineered waterfall data (see below).
3. Number of responses assumed in the analysis for the PDT: The number of responses reported in the article that were assumed for the analysis of the data for the PDT, by arm. As above, this was extracted for comparison with the reverse engineered waterfall data.
4. Reported point estimate for the PDT: If given, the standard point estimates presented in the article were extracted by arm.
5. Reported confidence interval for the PDT: If given, the standard confidence interval presented in the article was extracted by arm.
6. Reverse engineered PSA change data: The WebPlotDigitizer tool was used on each waterfall plot to reverse engineer the raw continuous data upon which the waterfall plot was based, for subsequent re-analysis. This tool in general provides high precision for reverse engineering of data, but some small inaccuracies are unavoidable. Consequently, we discuss later sensitivity analyses that were performed to assess the impact of any inaccuracies.
7. Disease population: To stratify our analyses later, information on the particular population eligible for the trial was extracted (e.g., mCRPC, localised PC).
8. Phase: For contextual information, the phase of the trial (e.g., phase I).
9. Number of clipped bars: A number of waterfall plots clip the presentation of their data at an upper percentage increase in PSA from baseline. The number of bars that were clipped in this way was extracted to enable a sensitivity analysis to be performed to what the true value may have been.
10. Clip point: The (approximate) value at which any bars were clipped.

As noted in the main manuscript, some small differences in extracted data for the included articles in the pilot evaluation were present. These differences were a consequence of differential handling of Items 2-4 by the reviewers in scenarios where the waterfall data did not match the results given in the text. This occurred, e.g., due to the waterfall plot containing results only for those patients successfully evaluated for PSA change, while the analysis reported in the text assumed a sample size based on all enrolled patients. As the purpose of manually extracting Items 1-5 and 9-10 was to perform an automated comparison with the reverse engineered data, to establish successful use of the WebPlotDigitizer tool and not to evaluate quality of data extraction, the remaining 144 articles were randomly allocated for single review between JMSW, MJG, and MMM.

Overall analysis

In the main manuscript results are presented for the re-analysis of 78 reverse engineered mCRPC datasets. Here, we present the findings based on the re-analysis of all extracted 121 datasets. Results are given in Figure S1.

In all 121 datasets, the augmented analysis approach returned a 95% CI with a narrower width (Figures S1B and S1C). The median efficiency gain from using the augmented analysis, in terms of the percentage reduction in the width of the 95% confidence interval for the response rate, was 24.9% (IQR [18.3%,42.4%]). In terms of the implied percentage increase to the original sample size (Figure S1D), this translates to a median efficiency gain of 107.6% (IQR [89.1%,220.9%]).

Sensitivity analyses

The results of the performed sensitivity analyses are presented below.

To evaluate whether the augmented approach is robust to the precise value of the underlying continuous data, which would provide reassurance on the employed reverse engineering procedure, 100 replicate re-analyses for each of the 121 included arms were performed. In each replicate, random noise uniformly distributed on [-1,1] was added to each continuous outcome. The augmented analysis procedure was then utilized and the efficiency gains determined. Boxplots of the efficiency gains are shown for each of the 121 arms in Figure S2. For a small number of trials, small perturbations do result in large changes in efficiency. Routinely, this is a consequence of the initial trial having a very low sample size and/or standard point estimate for the PSA response rate. For the majority of trials, however, introducing small random variation to the continuous data has little impact on the efficiency results.

To determine the potential impact of the clipped bars, for the 42 arms in which at least one bar was clipped, several multipliers were in turn applied to the clipped bar percentages before re-analysis (e.g., a multiplier of 2 for bars clipped at 100% means all bars in which the extracted data was 100% were changed to 200% before the analysis was performed). Increasing values of the multiplier then allow assessment of the degree to which the true percentage may have impacted the efficiency findings. The results of this analysis are shown in Figure S3. Whilst both measures of efficiency have the largest median for the original extracted data (i.e., a multiplier of 1), substantial efficiency gains were still observed for a multiplier of 10. This provides further assurance on the robustness of the results reported in the main manuscript.

**Figure S1:** Comparison of the standard and augmented analysis approaches for the 121 included PC datasets. Points are shaded according to the value of the standard point estimate. A: The standard and augmented point estimated are compared. B: The width of the standard and augmented point estimates are compared. C: The efficiency gains, in terms of the percentage confidence interval width reduction, are given. D: The efficiency gains, in terms of the percentage increase to the trial’s sample size, are given. For D, the limits are constrained to [0,500] for aesthetic purposes; 14 trials for which substantially larger efficiency gains were observed are omitted from this sub-figure.

**Figure S2:** The efficiency gain for the 121 included treatment arms across 100 replicate analyses in which random noise was added to the continuous data are shown through boxplots.

**Figure S3:** The median and IQR efficiency gain across 42 arms in which data was clipped is shown as a function of the multiplier applied to the clipped bars before re-analysis**.**
